# Consolidation immunotherapy following concurrent chemoradiotherapy in a patient with sinonasal NUT carcinoma: a case report

**DOI:** 10.3389/fonc.2024.1368187

**Published:** 2024-12-06

**Authors:** Xiaotao Geng, Xiaolong Chang, Xiaoli Wang, Shunjia Li, Guiyan Han, Zhiyu Song, Furong Hao, Jianwen Li

**Affiliations:** ^1^ Department of Radiation Oncology, Weifang People’s Hospital, Weifang, China; ^2^ Department of Pathology, Weifang People’s Hospital, Weifang, China; ^3^ Department of Otolaryngology, Weifang People’s Hospital, Weifang, China

**Keywords:** NUT, sinonasal, concurrent chemoradiotherapy, immunotherapy, PD-1 inhibitor

## Abstract

**Background:**

Nuclear protein in testis (NUT) cancers, also known as midline cancers, tends to occur in organs near the midline, such as the nasal sinuses and mediastinum. NUT carcinoma is very rare and has a poor prognosis.

**Case description:**

We report the case of a 44-year-old female patient with sinonasal NUT carcinoma who presented with a soft tissue mass in the left frontal sinus, ethmoid sinus, and left nasal cavity on computed tomography; the tumor was poorly demarcated from the left rectus medialis. After discussion with a multidisciplinary team with expertise on head and neck tumors, the patient was considered inoperable, and definitive concurrent chemoradiotherapy (CCRT) was recommended. The patient underwent CCRT followed by three cycles of consolidation chemotherapy with albumin-bound paclitaxel and nedaplatin. Subsequently, the patient underwent 16 cycles of consolidation therapy with the programmed death–1 (PD-1) inhibitor tislelizumab. The immune-related adverse events included grade 2 hypothyroidism. After CCRT, consolidation chemotherapy, and consolidation immunotherapy, the patient achieved a favorable outcome. The patient survived for 31 months, and there were no signs of recurrence or metastasis during follow-up.

**Conclusion:**

At present, there is no clear consensus on the consolidation treatment plan after CCRT for sinonasal NUT cancer. We used consolidation immunotherapy for the first time and achieved good efficacy, providing an innovative and promising treatment plan for refractory sinonasal NUT cancer.

## 1 Introduction

Nuclear protein in testis (NUT) carcinoma is extremely rare and has mostly been reported in case studies; the main primary tumor sites are the chest and head and neck (1, 2). The main molecular feature is a rearrangement of the testicular nucleoprotein gene (*NUTM1*). Although *NUTM1* can fuse to numerous different partner genes, it most frequently forms a *BRD4-NUTM1* fusion oncogene related to NUT carcinoma (2). The prognosis is very poor, with a median survival of less than 1 year (3). The treatment options for NUT cancer include surgery, radiation therapy, and chemotherapy. Surgery is critical for the treatment of NUT cancer, and surgery combined with postoperative chemoradiotherapy or radiotherapy is associated with improved survival (4). For patients with inoperable tumors or those who refuse surgery, radical concurrent chemoradiotherapy (CCRT) is an alternative. Recent advances in treatment options for head and neck NUT cancer include induction chemotherapy, proton radiotherapy, and immunotherapy. The SINTART 1 study showed that patients with tumor shrinkage greater than or equal to 80% after induction chemotherapy for surgically resectable sinonasal tumors were given the option of radiotherapy and exemption from surgical treatment (5). The SINTART 2 study showed that the addition of induction chemotherapy to treatment regiments for inoperable sinonasal tumors did not significantly improve survival (6). The above two phase II studies of induction chemotherapy did not include NUT cancers, and only one retrospective study of NUT cancers has analyzed the value of induction chemotherapy. Ramesh et al. (7) conducted a retrospective analysis of 12 patients with sinonasal NUT cancer and concluded that induction chemotherapy may be beneficial to patients. Patients with recurrent sinonasal NUT may be considered for proton radiotherapy. Muramatsu et al. (8) reported a case of sinonasal NUT carcinoma with local recurrence followed by reirradiation using proton radiotherapy, which led to complete response. In recent years, the rapid development of immunotherapy has led to the development of new options and useful additions to treatments for NUT cancer, which is often refractory. Currently, treatment with PD-1 and programmed death–ligand 1 (PD-L1) inhibitors has been reported for a small number of patients with lung, thyroid, and parotid NUT cancer (9, 10) but has not yet been reported for sinonasal NUT cancer. Moreover, there is no standard for consolidation regimens after CCRT. This study aimed to explore new treatment options for NUT cancer and strategies for consolidation immunotherapy after CCRT: we report the treatment of one patient with sinonasal NUT cancer with immunotherapy with PD-1 inhibitors after CCRT.

## 2 Case presentation

**Figure 1:**
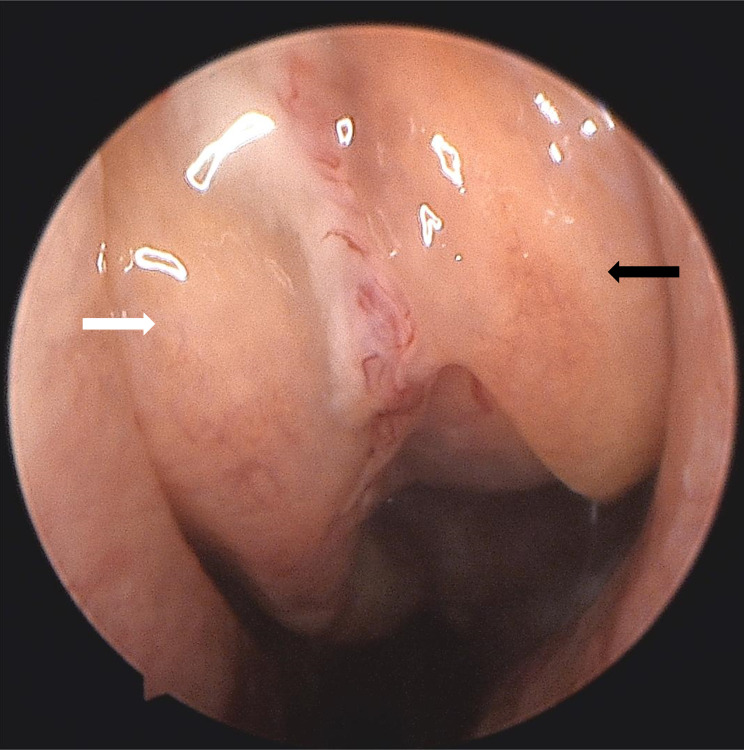
Nasal endoscopy revealed a mass (black arrow) in the left middle nasal meatus adjacent to the left middle turbinate (white arrow).

**Figure 2:**
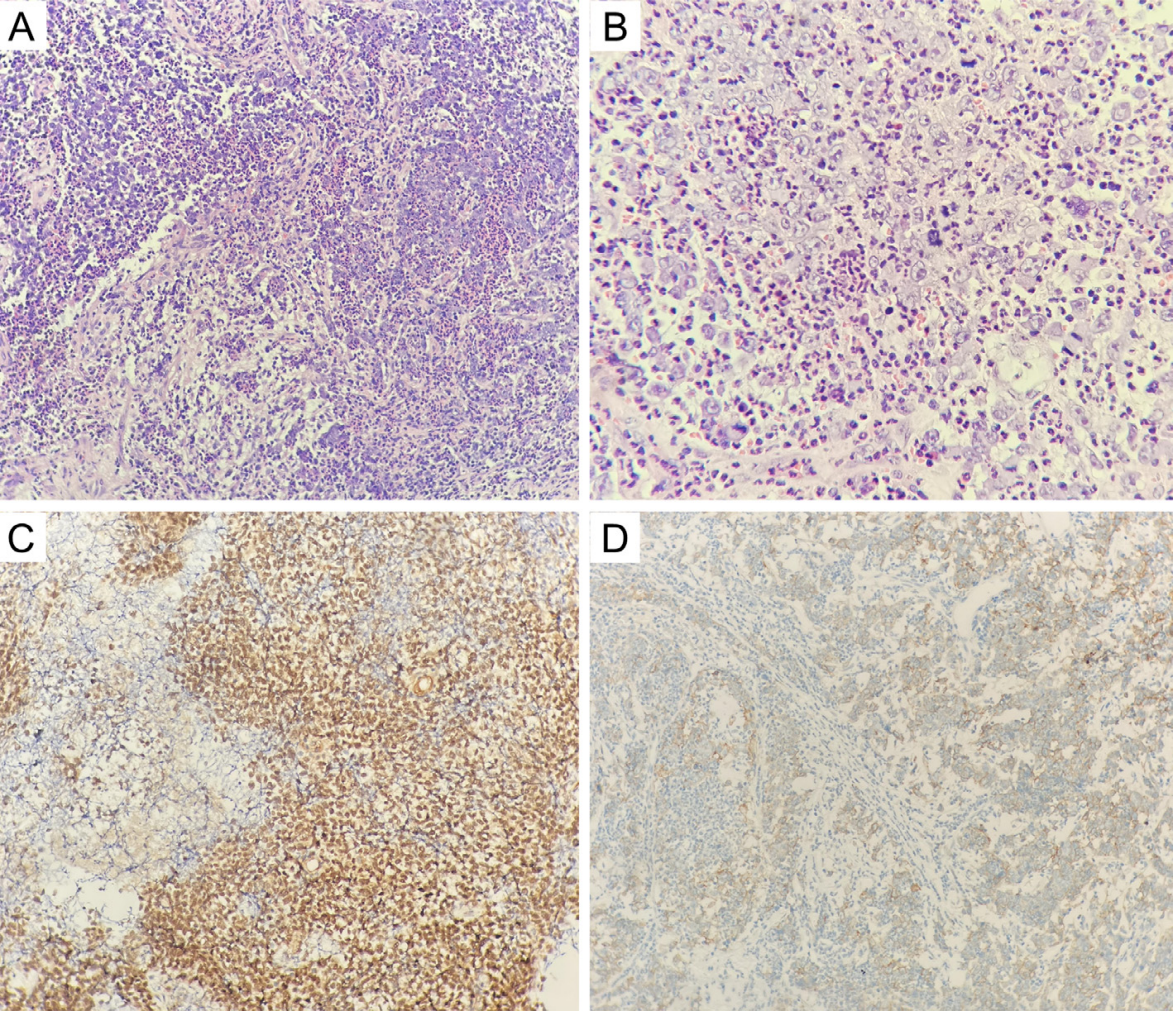
**(A, B)** The tumor cells were blue rounded heterogeneous cells, some of which were naked nucleated cells with minimal cytoplasm (hematoxylin and eosin; original magnification, ×200 and ×400); **(C)** positive NUT staining in the nucleus of tumor cells (hematoxylin and eosin; original magnification, ×200); **(D)** positive PD-L1 staining in tumor cells and immune cells (hematoxylin and eosin; original magnification, ×200).

**Figure 3:**
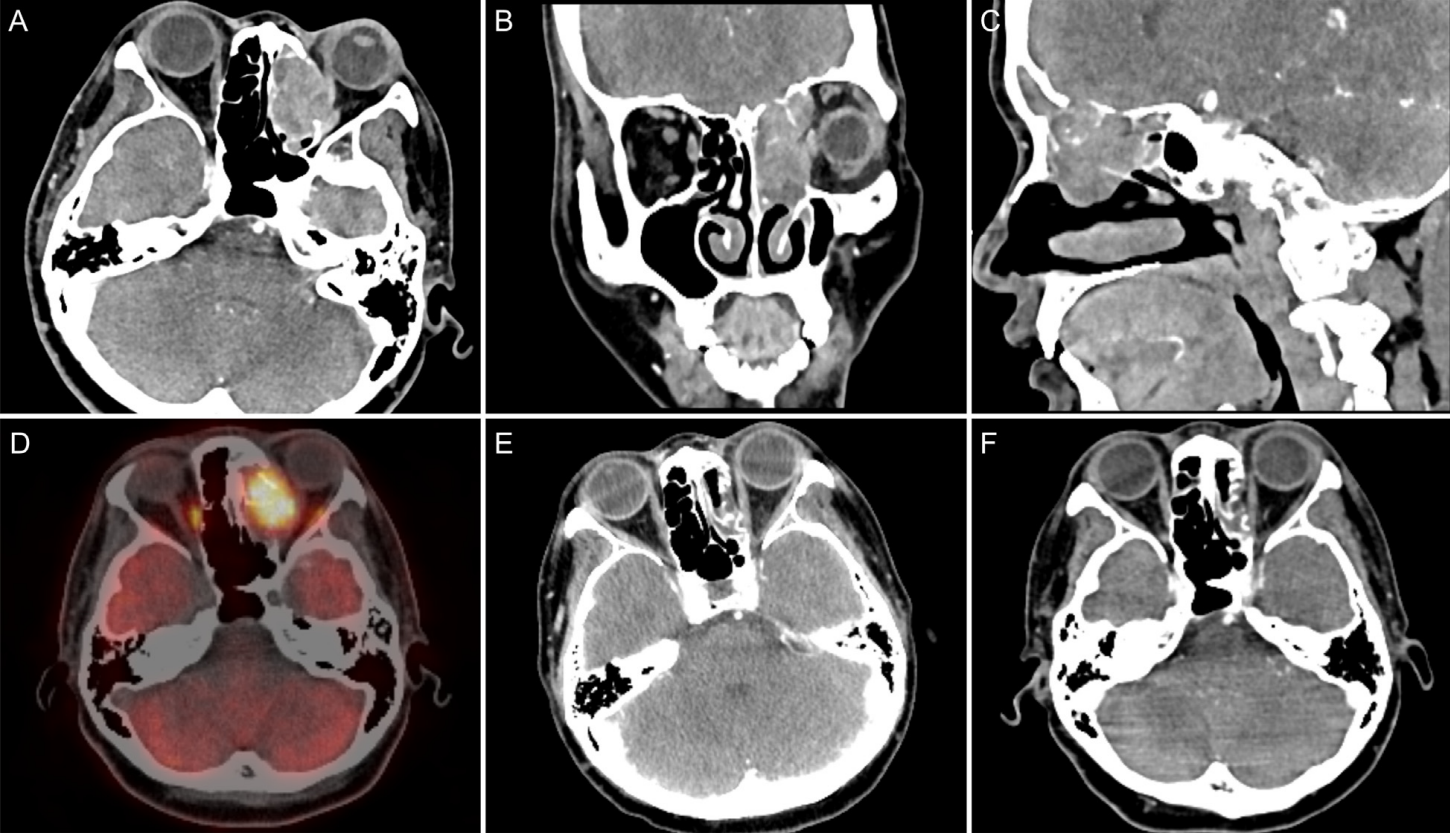
**(A)** CT axial view showed a mass in the left ethmoid sinus that was poorly demarcated from the left rectus medialis; **(B)** CT coronal view showed the mass invading the left orbit with intracranial invasion; **(C)** CT sagittal view shows the mass invading the frontal sinus with intracranial invasion; **(D)** PET/CT revealed a hypermetabolic mass with an SUVmax of 27.7 in the left ethmoid sinus; **(E)** CT after three cycles of consolidation chemotherapy revealed that the left ethmoid mass was considerably reduced compared with the previous mass, and the efficacy evaluation was PR; **(F)** the lesions reached a state of sustained remission after 13 cycles of immunotherapy, and the efficacy evaluation remained a PR.

**Figure 4:**
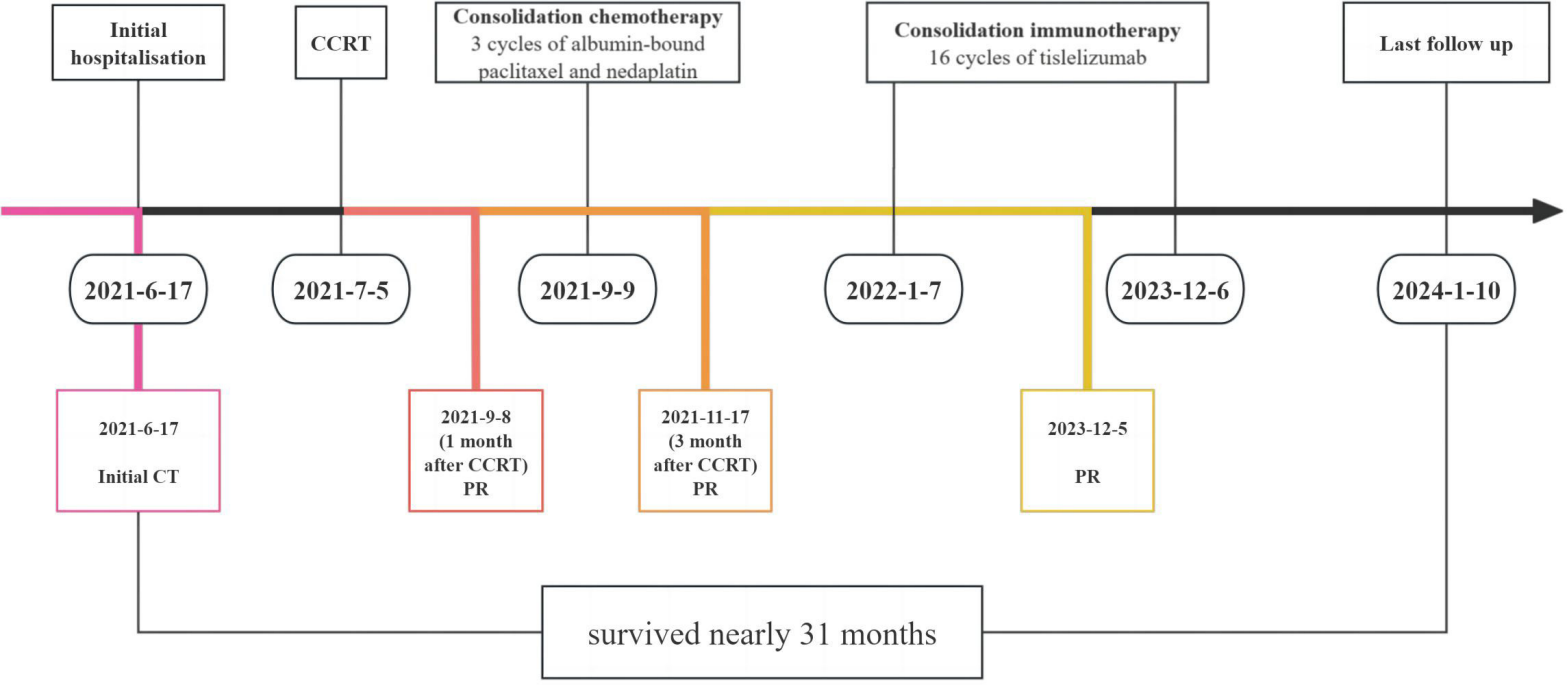
Timeline of the treatment. CCRT, concurrent chemoradiotherapy; PR, partial response.

The patient was a 44-year-old female from Shandong, China. She was first admitted to our hospital on 17 June 2021, with the complaint of left eye pain and headache for 3 months. She had undergone surgery for congenital heart disease 30 years prior. She was admitted to the hospital and underwent relevant examinations. Nasal endoscopy revealed a mass in the left middle nasal meatus adjacent to the left middle nasal turbinate (**Figure 1**). Biopsy pathology revealed that the tumor cells were blue, rounded, heterogeneous cells, some of which were naked nucleated cells with minimal cytoplasm (**Figures 2A, B**). Immunohistochemical (IHC) staining revealed tumor cells that were positive for NUT expression (**Figure 2C**). The Ki-67 mitotic index was nearly 70%. Epstein-Barr virus (EBV)-encoded RNA (EBER) *in situ* hybridization was negative. Based on NUT IHC, a diagnosis of NUT cancer was established. Further testing revealed positive PD-L1 expression in both tumor cells and immune cells (**Figure 2D**). The percentages of tumor cells and immune cells with PD-L1 positivity were 65% and 1%, respectively. Enhanced computed tomography (CT) of the sinuses revealed that most of the mass was located in the left ethmoid sinus, with the mass invading the frontal sinus upward, invading the medial orbital wall and the rectus medialis to the left, with a discontinuity of bone in the medial orbital wall on the left side of the orbital wall, invading the intracranial area upward, and breaching the wall of the floor of the sieve sinus downward into the middle nasal passages (**Figures 3A–C**). 18F-fluorodeoxyglucose (18F-FDG) PET/CT demonstrated hypermetabolic activity with increased fluorodeoxyglucose uptake in the ethmoid sinus mass (maximum standardized uptake value, SUVmax 27.7) (**Figure 3D**). Regional lymph node involvement and metastatic disease were also excluded. Considering the results of nasal endoscopy, paranasal sinus CT, and PET/CT, the final diagnosis was sinonasal NUT carcinoma (cT4bN0M0, stage IVA AJCC-8 version). After discussion with our multidisciplinary team (MDT) of experts on head and neck tumors, including the Department of Radiology, Pathology, Otolaryngology, Radiation Oncology and Medical Oncology, we concluded that the tumor was inoperable and recommended definitive CCRT. The patient agreed with the treatment plan derived from the MDT discussion and underwent definitive CCRT in our department. The gross tumor volume (GTV) contained macroscopic primary tumor detectable on CT imaging. The clinical target volume (CTV) was defined as the GTV plus a 5- to 10-mm margin to encompass the sites of microscopic extension including bilateral parapharyngeal space, bilateral retropharyngeal lymph node drainage area, left II–IV lymph drainage area, right partial II area, left cavernous sinus, bilateral sieve sinus and pterygoid sinus, frontal sinus, left maxillary sinus, nasopharyngeal, oropharyngeal, bilateral intrinsic nasal cavities, cranial base, bilateral pterygoid plate, pterygoid, medial pterygoid muscle, and pterygopalatine fossa. A planning target volume (PTV) was generated by incorporating a three-dimensional margin of 3 mm to the target volume in order to account for the uncertainties associated with treatment setup and internal organ mobility. The prescribed doses were 70 Gy and 60.06 Gy in 33 fractions, for the PTVs derived from GTV and CTV, respectively. Volume-modulated arc therapy technology was used to administer radiotherapy. During radiotherapy, the patient received three cycles of synchronized cisplatin chemotherapy (50 mg/m^2^ days 1–2 q3w) and sodium glycididazole (1.25 g days 1, 3, and 5 qw). According to the Response Evaluation Criteria in Solid Tumors, the efficacy of chemoradiotherapy (1 month after CCRT) was evaluated as a partial response (PR). Afterward, the patient received three cycles of consolidation chemotherapy with albumin-bound paclitaxel (260 mg/m^2^ day 1 q3w) and nedaplatin (80 mg/m^2^ day 1 q3w). The efficacy of three cycles of consolidation chemotherapy was evaluated as PR (**Figure 3E**). Considering that NUT cancer is a highly malignant tumor with a poor prognosis, subsequent consolidation immunotherapy was agreed upon after thorough communication with the patient. Sixteen cycles of consolidation therapy with intermittent tislelizumab (200 mg day 1) were started on 7 January 2022, and the last immunotherapy treatment was given on 6 December 2023. Details of the timing of the use of tislelizumab are given in the **Supplementary File**. After 16 rounds of immunotherapy, the ethmoid sinus lesions achieved a state of sustained remission, and the efficacy assessment revealed a PR (**Figure 3F**). Adverse effects throughout treatment are tolerable. Acute toxicity during radiotherapy is mainly characterized by localized radiodermatitis of the facial skin. According to the Radiation Therapy Oncology Group’s acute radiation morbidity scoring criteria for skin, acute radiation dermatitis was grade 1. There was no late toxicity after radiotherapy, and, to date, the patient has not experienced vision loss. The immune-related adverse events included grade 2 hypothyroidism. The recent workup on 6 December 2023 demonstrated no local recurrence or distant metastasis. The entire treatment timeline of the patient is shown in **Figure 4**.

## 3 Discussion

Sinonasal NUT carcinoma is very rare. There is a lack of large-scale epidemiologic studies and only a few retrospective studies with small sample sizes. A review of the literature by Lee et al. revealed that 4 of the 362 cases of poorly differentiated or undifferentiated carcinomas of the head and neck were sinonasal NUT carcinomas (11). A single-center study from China revealed that 3 of the 145 cases of sinonasal malignancies were NUT carcinomas (12). NUT has a very unfavorable prognosis. The median survival time for patients with primary NUT cancer of the chest is only 4.4 months (13). Compared to that for primary tumors in the lungs, the median survival time for primary NUT cancers of the head and neck is slightly greater, at only 9.7 months (13). In head and neck NUT cancers, survival may also vary depending on the location of the primary focus. A recent single-center study of 12 patients with sinonasal NUT carcinoma revealed median OS and median disease-specific survival times of up to 14.6 months (7).

Pathologic diagnosis of NUT cancer includes IHC and genetic testing, and genetic testing methods include fluorescence *in situ* hybridization (FISH), next-generation sequencing (NGS), and reverse transcription–polymerase chain reaction (RT-PCR) (14). Diffuse (>50%) positive NUT expression on IHC is sufficient for diagnosing NUT cancer (15). The diagnosis of NUTMI molecular rearrangements by FISH and NGS is not necessary, but the above two tests are helpful in determining the prognosis of NUT cancer patients (13). In this case, the patient was diffusely positive for NUT expression according to IHC, and the diagnosis was confirmed on this basis.

We reviewed the literature on sinonasal NUT cancer (7, 8, 12, 16–30) (**Table 1**). Treatment decisions were made on the basis of the above literature by first assessing the tumor stage. The treatments for locally advanced disease include surgery combined with postoperative adjuvant radiotherapy, definitive chemoradiotherapy, induction chemotherapy combined with surgery, and induction chemotherapy combined with radiotherapy. The treatments for metastatic disease include chemotherapy combined with immunotherapy and debulking surgery and chemotherapy combined with local palliative radiotherapy. Bromodomain and extra-terminal domain inhibitors may be an option after progression on first-line therapy for metastatic NUT cancer. In this case, tumor was considered late stage and considered to be unsuitable for surgical treatment by the MDT; ultimately, CCRT was selected as the treatment strategy. After CCRT, we first performed consolidation chemotherapy include albumin-bound paclitaxel and nedaplatin. There are few previous studies on consolidation chemotherapy after CCRT for sinonasal cancer. The consolidation chemotherapy regimen can refer to the induction chemotherapy regimen. The most commonly used induction chemotherapy regimens for sinonasal cancer are docetaxel and platinum (TP) and docetaxel, cisplatin, and fluorouracil (TPF) (31). TP includes paclitaxel and cisplatin, and TPF includes paclitaxel, cisplatin, and fluorouracil. These two consolidation chemotherapy regimens also provide a reference for consolidation chemotherapy for nasal sinus NUT cancer. At the end of CCRT and consolidation chemotherapy, the patient received consolidation immunotherapy based on three considerations. First, recent evidence has confirmed that consolidation therapy with immune checkpoint inhibitors (ICIs) can improve the survival of patients with lung and esophageal cancer after CCRT (32–34). In rare tumors, such as pulmonary sarcomatoid carcinoma (PSC) and pulmonary pleomorphic carcinoma (PPC), there are also case reports of encouraging results with consolidation immunotherapy after CCRT (35, 36). Although there is currently no evidence of survival benefits from ICI consolidation therapy after CCRT in patients with head and neck tumors, these findings in patients with lung and esophageal cancer can guide studies on head and neck tumors. A summary of clinical trials or case reports of consolidation immunotherapy after radiotherapy for malignant tumors is listed in **Table 2** (32, 35–42). These clinical trials and case reports provide some reference for the selection of future consolidation ICI regimens after chemoradiotherapy for sinonasal NUT cancer patients. Second, although the patient’s treatment efficacy after radiotherapy was evaluated as PR, NUT cancer has a poor prognosis and is prone to recurrence and metastasis, and good recent treatment efficacy may not necessarily indicate a good long-term prognosis. Thus, maintenance therapy may be needed after radical treatment is completed. Maintenance therapy requires the selection of an agent that is both highly effective and less toxic, and PD-1 inhibitor immunotherapy may be an option. Third, the case in this study had high PD-L1 expression in tumor cells. High-dose anti–PD-1/anti–PD-L1 therapy is generally believed to indicate a greater response rate and clinical benefit when PD-L1 is expressed (43). According to a recent report from the NUT symposium, patients with PD-L1 positivity or a high tumor mutation load can receive ICIs in combination with chemotherapy (15). Additionally, there have been recent case reports of the combined use of immunotherapy in head and neck NUT cancers. One patient had thyroid NUT cancer combined with carelizumab immunotherapy in addition to postoperative chemotherapy (9). Another case involved parotid NUT cancer, which was treated with targeted agents combined with sintilimab immunotherapy after multiple postoperative metastases were detected (10).

**Table 1:** Summary of previous studies reporting sinonasal NUT cancer.

Authors	Year	Number of patients	Location	Treatment	Outcome
Ramesh et al. (7)	2024	12	Nasal cavity maxillary sinus ethmoid sphenoid	Surgery ± induction chemotherapy; chemoradiotherapy ± induction chemotherapy	14.6 months (median OS)
Caner et al. (16)	2024	1	Nasal cavity	Induction chemotherapy combined with immunotherapy + surgery + radiotherapy	5.4 months (OS)
Qayum et al. (17)	2024	1	Maxillary sinus	Endoscopic sinus surgery+ maxillectomy	NM
Arai et al. (18)	2024	1	Maxillary sinus	Induction cheomotherapy + chemoradiotherapy (initial treatment); immunotherapy + targeted therapy and chemotherapy (after progression)	11 months (OS)
Wang et al. (12)	2023	3	Nasal cavity maxillary sinus ethmoid	Surgery + radiotherapy	One patient 13 months (OS); two survived 12 and 15 months
Wartenberg et al. (19)	2023	1	Maxillary sinus	Surgery + radiotherapy	NM
da Costa et al. (20)	2023	1	Nasal cavity	Chemotherapy	NM
Zheng et al. (21)	2023	1	Maxillary sinus	Chemoradiotherapy + chemotherapy	NM
Wei et al. (22)	2022	1	Nasal sinus; frontal sinus	Surgery + radiotherapy	NM
Muramatsu et al. (8)	2022	1	Ethmoid	Chemoradiotherapy	survived 26 months
Patel et al. (23)	2021	1	Sphenoid	Ehemotherapy + radiotherapy (initial treatment); targeted therapy (after progression)	survived 21 months
Vakani et al. (24)	2020	1	Sphenoid	NM	NM
Oliveira et al. (25)	2019	1	Maxillary sinus	Induction cheomotherapy + chemoradiotherapy; chemotherapy (after progression)	NM
Arimizu et al. (26)	2018	1	Nasal cavity	Chemotherapy + chemoradiotherapy + surgery; chemotherapy + targeted therapy (after progression)	9 months (OS)
Edgar et al. (27)	2017	1	Nasal cavity	Surgery + radiotherapy and chemotherapy	3 months (OS)
Yang et al. (28)	2015	1	Nasal cavity	Surgery + radiotherapy and chemotherapy	Survived 10 months
Suzuki et al. (29)	2014	1	Nasal cavity	Chemoradiotherapy	Survived 12 months
Hsieh et al. (30)	2011	1	Nasal cavity	Chemoradiotherapy	NM

NM, not mentioned.

**Table 2:** Summary of clinical trials or case reports of consolidation ICI after chemoradiotherapy in patients with different cancer.

Trial/NCT number/authors	ICI	Target	Dose	Frequency	Duration (time/cycles)	Cancer type
RATIONALE 311 (37)	Tislelizumab	PD-1	200 mg	q3w	24 months	ESCC
Geng et al. (our case)				Irregular	23 months	Sinonasal NUT cancer
NCT03671265 (38)	Camrelizumab	PD-1	200 mg	q2w	32 weeks (from the beginning of radiotherapy)	ESCC
InTRist (39)	Toripalimab	PD-1	240 mg	q3w	12 months or until progression	NSCLC
KEYNOTE-412 (40)	Pembrolizumab	PD-1	200 mg	q3w	14 cycles	HNSCC
PACIFIC (32)	Durvalumab	PD-L1	10 mg/kg	q2w	12 months or until progression	NSCLC
ADRIATIC (41)			1,500 mg	q4w	24 months or until PD or intolerable toxicity	SCLC
Wang et al. (36)			620 mg	q2w	12 months	PSC
Yorozuya et al. (35)			NM	NM	12 months	PPC
JAVELIN Head and Neck 100 (42)	Avelumab	PD-L1	10 mg/kg	q2w	12 months	HNSCC
NCT03377400 (34)	Durvalumab	PD-L1	1,500 mg	q4w	24 months	ESCC
	Tremelimumab	CTLA4	75 mg			

ICI, immune checkpoint inhibitor; NCT, National Clinical Trial; PD-1, programmed cell death–1; PD-L1, programmed cell death–ligand 1; NM, not mentioned; q3w, every 3 weeks; q2w, every 2 weeks; q4w, every 4 weeks; CTLA4, cytotoxic T-lymphocyte antigen–4; ESCC, esophageal squamous cell carcinoma; NSCLC, non–small-cell lung cancer; SCLC, small cell lung cancer; HNSCC, head and neck squamous cell carcinoma; PSC, pulmonary sarcomatoid carcinoma; PPC, pulmonary pleomorphic carcinoma.

## 4 Conclusions

NUT carcinoma of the nasal cavity and sinuses is very rare, and only 50 cases have been reported in the literature. Surgery is the first choice for the treatment of NUT carcinoma, and CCRT can be chosen for patients who cannot be treated surgically. Compared with surgery, radiotherapy has the advantage of preserving organ function, thus improving the quality of life of patients. There is no consensus yet on consolidation treatment after CCRT. In this case, we studied a case of ethmoid NUT cancer that was treated with consolidation PD-1 inhibitor therapy after CCRT, which yielded a good therapeutic response. More case studies are needed in the future to validate the efficacy of consolidation immunotherapy after CCRT and to study the underlying mechanisms involved.

## Data Availability

The original contributions presented in the study are included in the article/**Supplementary Material**. Further inquiries can be directed to the corresponding author.
