# Supplementary material for: Consolidation immunotherapy following concurrent chemoradiotherapy in a patient with sinonasal NUT carcinoma: a case report
Source: Front Oncol. 2024 Dec 6;14:1368187. doi: 10.3389/fonc.2024.1368187 (PMC11659667; doi:10.3389/fonc.2024.1368187)
Supplement: Supplementary file 1 [file Table1.docx]

Supplementary Material

Table1| The timing of the use of tislelizumab

| **Time** | **PD-1 inhibitor** | **Dose** | **Cycle** |
| --- | --- | --- | --- |
| 2022-1-7 | tislelizumab | 200mg | 1 |
| 2022-2-17 | tislelizumab | 200mg | 2 |
| 2022-4-19 | tislelizumab | 200mg | 3 |
| 2022-6-2 | tislelizumab | 200mg | 4 |
| 2022-7-12 | tislelizumab | 200mg | 5 |
| 2022-8-12 | tislelizumab | 200mg | 6 |
| 2022-9-6 | tislelizumab | 200mg | 7 |
| 2022-11-9 | tislelizumab | 200mg | 8 |
| 2023-2-10 | tislelizumab | 200mg | 9 |
| 2023-3-9 | tislelizumab | 200mg | 10 |
| 2023-4-17 | tislelizumab | 200mg | 11 |
| 2023-5-24 | tislelizumab | 200mg | 12 |
| 2023-7-18 | tislelizumab | 200mg | 13 |
| 2023-8-23 | tislelizumab | 200mg | 14 |
| 2023-11-7 | tislelizumab | 200mg | 15 |
| 2023-12-6 | tislelizumab | 200mg | 16 |
